# Efficacy of a third-generation oncolytic herpes simplex virus in neuroendocrine tumor xenograft models

**DOI:** 10.18632/oncotarget.27391

**Published:** 2019-12-24

**Authors:** Hideyuki Matsushima, Masaki Kaibori, Masahiko Hatta, Morihiko Ishizaki, Richi Nakatake, Tadayoshi Okumura, Kengo Yoshii, Tomoki Todo

**Affiliations:** ^1^Department of Surgery, Kansai Medical University, Hirakata, Osaka, Japan; ^2^Research Organization of Science and Technology, Ritsumeikan University, Kusatsu, Shiga, Japan; ^3^Department of Mathematics and Statistics in Medical Sciences, Kyoto Prefectural University of Medicine, Kyoto, Japan; ^4^Division of Innovative Cancer Therapy, Advanced Clinical Research Center, Institute of Medical Science, The University of Tokyo, Minato-ku, Tokyo, Japan

**Keywords:** oncolytic virus, herpes simplex virus, neuroendocrine tumor, mouse xenograft model

## Abstract

BACKGROUND: Few chemotherapies are available for neuroendocrine tumors, especially for highly malignant neuroendocrine cancers. The third-generation oncolytic herpes simplex virus type 1 (HSV-1) T-01 selectively replicates in tumor cells and shows cytotoxicity against tumor cells without damaging surrounding normal tissues. We examined the antitumor effect of T-01 to explore novel treatments for patients with neuroendocrine tumors.

METHODS: The cytotoxicity of T-01 was tested in two human and one murine neuroendocrine tumor cell lines *in vitro*. Mouse models with subcutaneously implanted human neuroendocrine tumor QGP1 cells were used to investigate T-01 efficacy *in vivo*.

RESULTS: T-01 showed cytotoxicity against the three cell lines *in vitro*. In xenograft models, the growth of tumors derived from QGP1 cells was inhibited by T-01 compared with control group. Although weight loss of mice was observed with tumor growth in the control group, it was suppressed by T-01 administration. The antitumor effects of T-01 were dependent on virus concentration and frequency of administration.

CONCLUSIONS: T-01 effectively inhibits tumor cell proliferation in a poorly differentiated NEC mouse model. These results suggest that the third-generation oncolytic HSV-1 may serve as a novel treatment for patients with neuroendocrine tumors.

## INTRODUCTION

Neuroendocrine tumor (NET), which originates from neuroendocrine cells, is a rare tumor occurring in 3–5 individuals per 100,000 people a year. Many NETs occur in the pancreas and gastrointestinal tract, and liver metastases are frequently observed [1]. The World Health Organization (WHO2017) has defined and classified NET [2]. Among NETs, neuroendocrine carcinoma (NEC) shows poor differentiation, and high-grade NEC is characterized by metastasis or recurrence with poor prognosis. Few effective chemotherapy treatments are available for NEC patients, although cisplatin-based therapy can be effective. However, secondary treatment is not available for patients with poor response to cisplatin-based treatments [3, 4].

Some studies have indicated that oncolytic herpes simplex virus type 1 (HSV-1), which selectively replicates in and damages tumor cells, may be effective for treating NETs. HSV-1 infects a variety of cell types and causes strong cytotoxicity; its cell-to-cell spread is not affected by circulating antibodies and antiviral drugs are available, indicating HSV-1 is suitable for clinical application [5].

Oncolytic HSV-1 (oHSV) uses features of the HSV-1 life cycle to specifically destroy tumor cells without harming normal cells. Mutations in viral genes associated with pathogenicity, viral DNA synthesis, or both can limit viral replication in tumor cells [6]. Cross-resistance to other therapeutic strategies such as chemotherapy does not occur [6]. The key to developing a useful oHSV is to achieve high antitumor efficacy without compromising safety.

oHSV is under Phase I to III clinical trials for treatment for solid cancer [7–11]. The oHSV G207 mutant, derived from HSV-1 strain F, has deletions in both copies of the γ34.5 gene and a lacZ insertion that inactivates the ICP6 gene, which permits replication in cancer cells that can complement these mutations, but not in normal cells, including neurons [12]. The mutant G47Δ was derived from G207 by introducing an additional deletion within the α47 gene that overlaps the US11 promoter [13]. Compared with G207, G47Δ replicates more efficiently and increases the presentation of the MHC class I molecule while maintaining the safety profile of G207 [13]. These properties led to an enhanced cytotoxic lymphocyte response against tumor cells and enhanced therapeutic efficacy of the virus, as indicated by the results from animal models of brain tumors, prostate cancer, breast cancer, and neurofibroma [13–16]. In Japan, a clinical trial of G47Δ in patients with recurrent glioblastoma, prostate cancer, or olfactory neuroblastoma is underway [17].

T-01 has a genomic structure similar to that of G47Δ [18]. The α47 and γ34.5 loci are deleted from the HSV-1 genome, and the LacZ gene replaces the ICP6 gene. We recently demonstrated that T-01 effectively inhibited the growth of human hepatocellular carcinoma and hepatoblastoma in mouse models [19].

In this study, we evaluated the potential anti-tumor activity of T-01 in NET using NET cell lines and a mouse tumor xenograft model.

## RESULTS

### Cytopathic effects of T-01 and virus yields *in vitro*

**Figure 1:**
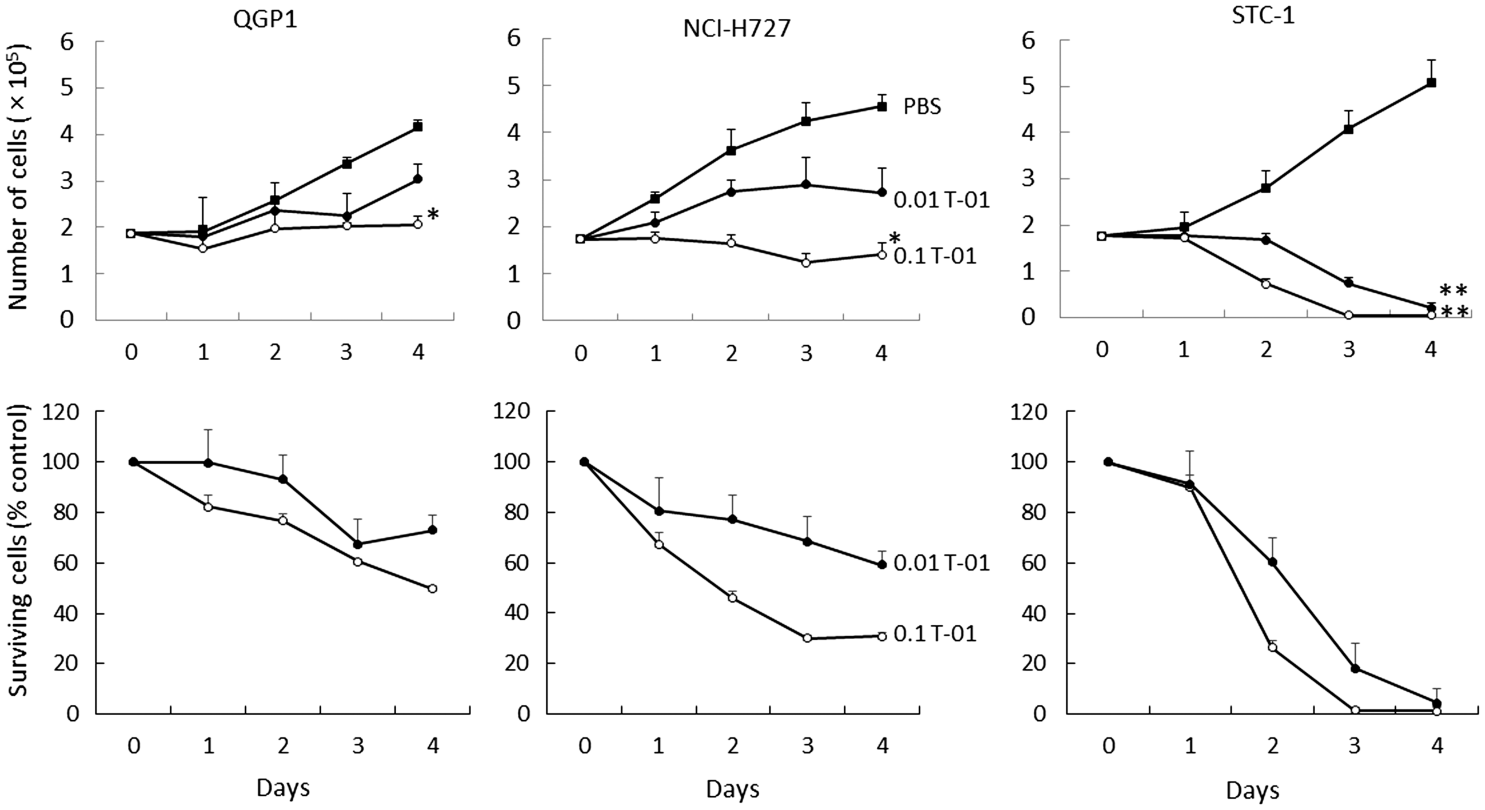
Cytotoxic activity of T-01 *in vitro*. Cell lines (QGP1, NCI-H727 and STC-1) were treated with T-01 virus (MOI = 0.01 (filled circles) or 0.1 (open circles)) or PBS (filled squares) and incubated for the indicated days. The number of surviving cells was counted (top row) and the percentage compared with PBS controls was determined (bottom row) at each time point. Data represent mean ± SE (*n* = 6/time point). ^*^ *P* < 0.05 and ^**^ *P* < 0.01 vs. PBS treatment.

We first examined the effects of T-01 on human pancreatic neuroendocrine carcinoma cells (QGP1), human pulmonary NET cells (NCI-H727) and murine NET cells (STC-1) using *in vitro* cytotoxicity assays (*n* = 6 per group). Infection with T-01 resulted in a cell proliferation inhibition on days 1–4 (Figure 1). Infection at a high MOI (MOI 0.1) significantly decreased the number of QGP1, NCI-H727 and STC-1 cells, resulting in 50% (*P* < 0.001), <40% (*P* < 0.001) and <10% (*P* < 0.001), respectively, compared with controls (phosphate buffered saline, PBS) on day 4. Infection at a low MOI (MOI 0.01) decreased the numbers of QGP1, NCI-H727 and STC-1 cells to 80%, 60% and <10% at day 4, respectively. Although slow proliferation of infected QGP1 and NCI-H727 cells was observed, there was a tendency of T-01-mediated inhibition of proliferation in these cells.

**Figure 2:**
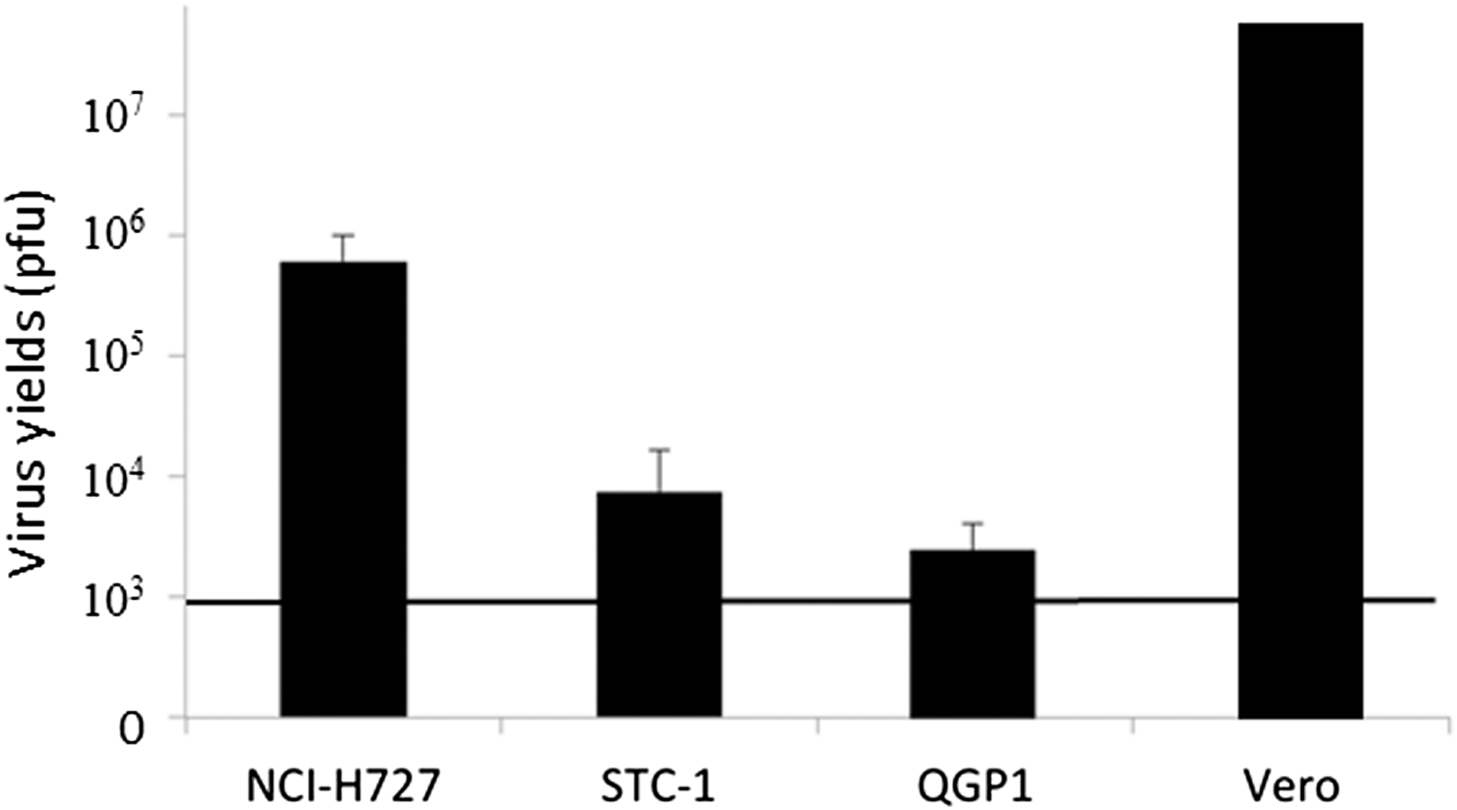
Virus replication of T-01 *in vitro*. *In vitro* virus yields were determined using plaque assays 48 h after infection with T-01 (MOI = 0.01) in Vero or neuroendocrine tumor cells (NCI-H727, STC-1 and QGP1) (5 × 10^5^ cells per well). The bold line indicates the initial virus concentration. Data represent mean ± SE (*n* = 9).

In examining virus replication capacity at a low MOI (0.01), the *in vitro* virus yield was measured using the puller assay after initial infection at a virus concentration of 5.0 × 10^3^ pfu and 48 h culture. The virus concentrations increased to 13-, 6.6- and 1.5-fold in NCI-H727, STC-1 and QGP1 cells, respectively (Figure 2). T-01 showed good replication capabilities in these cultured cell lines.

### Effects of T-01 in mice with subcutaneous tumors

**Figure 3:**
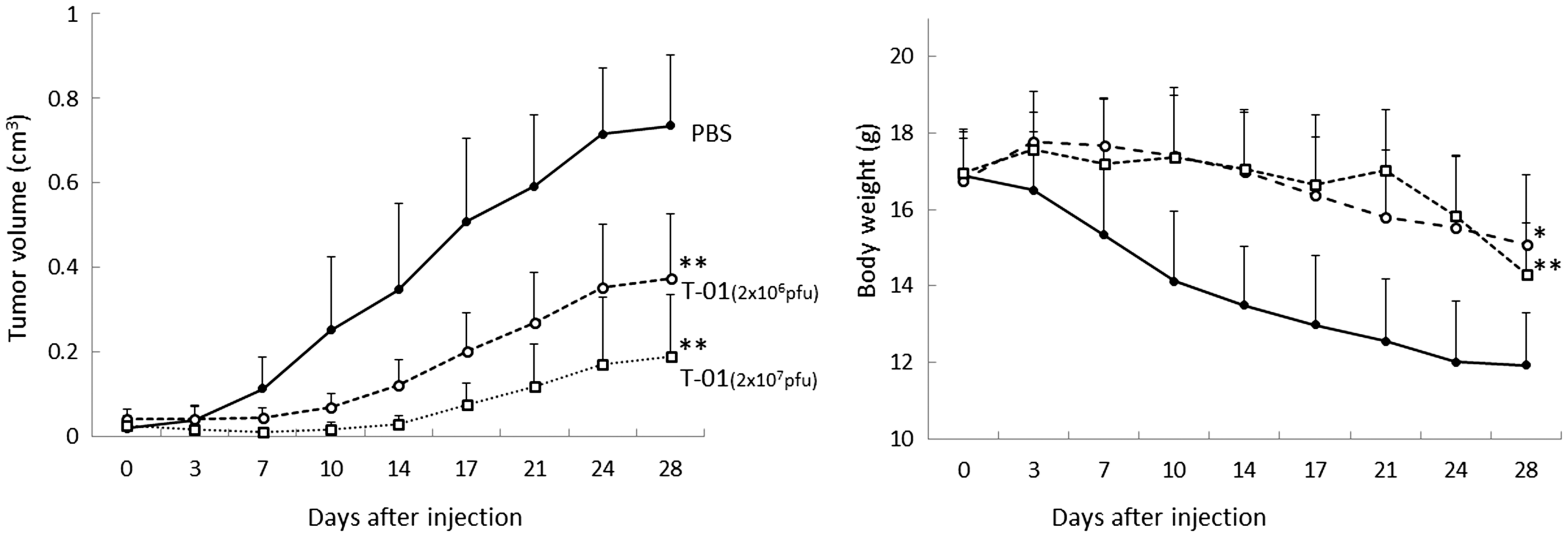
Antitumor effect of T-01 in a dose-dependent manner in mice with subcutaneous tumors. QGP1 cells were implanted subcutaneously in female athymic mice. Tumors were inoculated twice (days 0 and 3) with T-01 (2 × 10^6^ pfu (open circle) or 2 × 10^7^ pfu (open squares)) or PBS (solid circles). Tumor growth (left) and body weight (right) were monitored. Data represent mean ± SE (*n* = 8 mice/group). ^*^ *P* < 0.05 and ^**^ *P* < 0.01 vs. PBS treatment.

We next examined the effects of T-01 in an athymic mouse model with subcutaneous tumors generated with QGP1 human NEC cells. When subcutaneous tumors were palpable 7 days after transplantation, T-01 or PBS was inoculated twice on days 0 and 3, since a single treatment at day 0 was less effective than two inoculations as shown in our previous study [19]. We evaluated three treatment groups: PBS, 2.0 × 10^6^ and 2.0 × 10^7^ pfu (*n* = 8 per group) (Figure 3). Tumor growth was inhibited in both T-01 groups compared with the PBS group in a dose-dependent manner (Figure 3, left). As the tumor proliferated, weight loss was observed in the PBS group. However, the T-01 administration groups only showed moderate weight loss (Figure 3, right).

**Figure 4:**
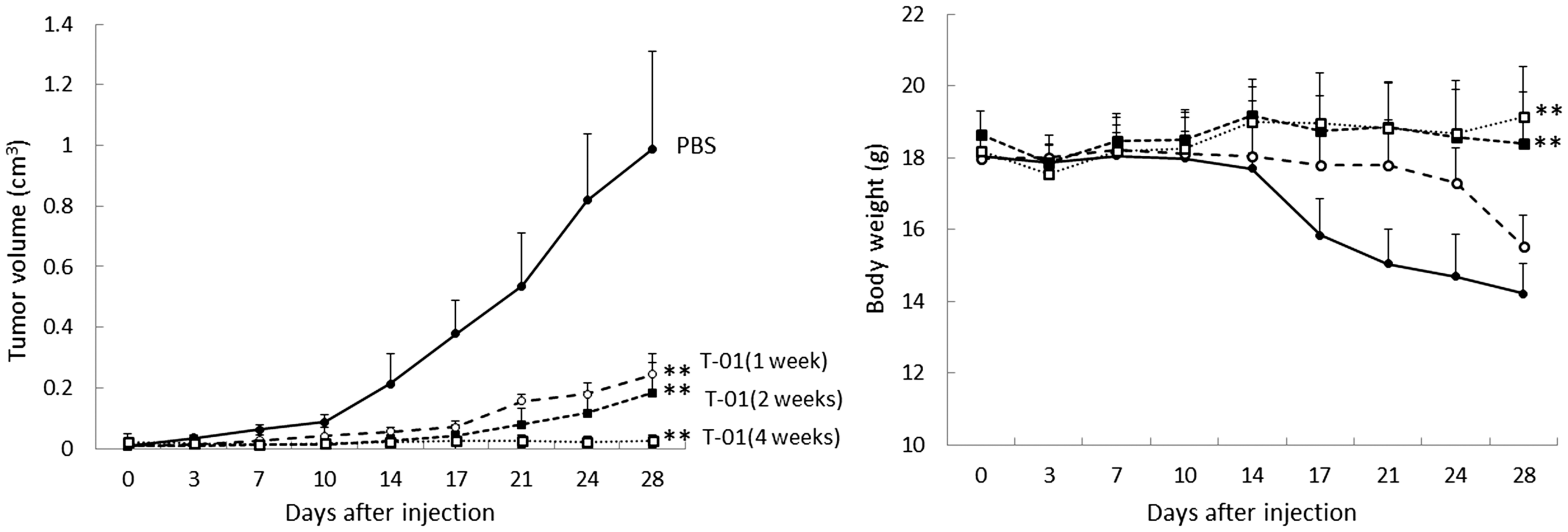
Antitumor effect of T-01 using various administration protocols in mice with subcutaneous tumors. QGP1-derived tumors in female athymic mice were inoculated twice a week (days 0 and 3) with T-01 (2.0 × 10^7^ pfu) for various times (1 week (open circle), 2 weeks (filled squares), 4 weeks (open squares)) or PBS (filled circles). Tumor growth (left) and body weight (right) were monitored. Data represent mean ± SE (*n* = 8 mice/group). ^*^ *P* < 0.05 and ^**^ *P* < 0.01 vs. PBS treatment.

We next examined tumor growth in response to various T-01 administration protocols (Figure 4). In one experiment, virus concentration was kept constant at 2.0 × 10^7^ pfu, and the tumor-bearing mice were infected with PBS or T-01 twice weekly for 1, 2 or 4 weeks (*n* = 8 per group). The tumor size tended to decrease more in the 2- and 4-week inoculation groups compared with the 1-week inoculation group (Figure 4, left). A significant tumor growth inhibitory effect was observed in each T-01 treatment group compared with the mock group on day 28: PBS, 0.989 ± 0.113 (cm^3^, tumor volume ± SE, *n* = 8); 1 week, 0.244 ± 0.014 (*P* < 0.001); 2 weeks, 0.183 ± 0.046 (*P* < 0.001); and 4 weeks, 0.025 ± 0.008 (*P* < 0.001). The tumor size of the 4-week administration group tended to be smaller than that of the 1-week administration group (*P* = 0.078).

Weight loss was observed in the 1-week inoculation group, but there was almost no weight loss in the 2- and 4-week administration groups (Figure 4, right).

These results suggest that the antitumor effects of T-01 depend on administration concentration and administration frequency.

### Hematologic and histological examination

**Figure 5:**
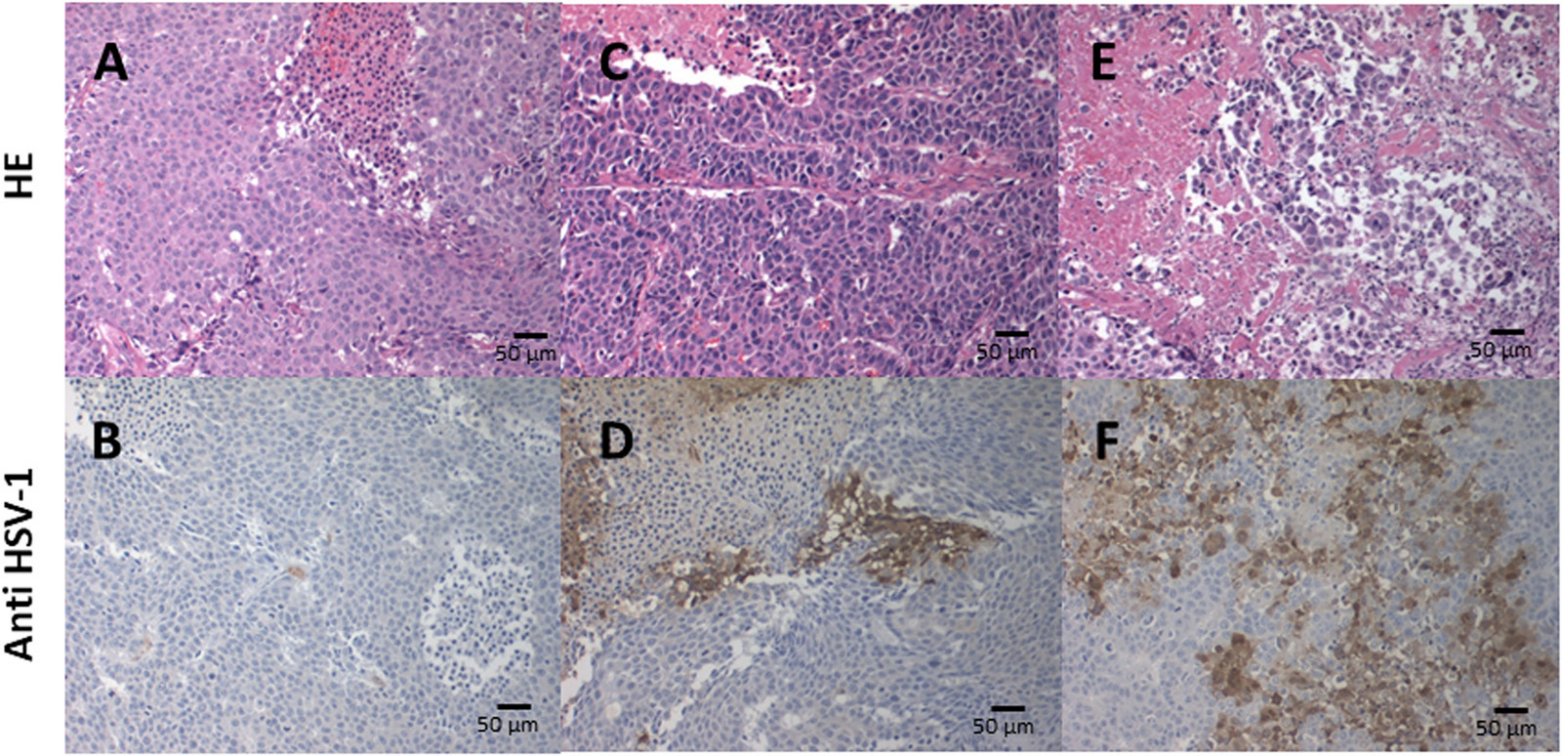
Immunohistochemical analysis of HSV-1 in xenograft tumors. Subcutaneous tumors were inoculated twice a week (days 0 and 3) for 1 and 4 weeks with T-01 (2 × 10^7^ pfu) or 4 weeks with PBS. Mice were sacrificed 28 days after inoculation and the tissue sections were stained with HE (upper; **A**, **C** and **E**) or anti-HSV-1 antibody (lower; **B**, **D** and **F**). Histological images of tumors with 4 weeks of PBS injection (A, B), 1 week of virus administration (C, D) and 4 weeks of virus administration (E, F). Representative images of three experiments are shown.

We performed pathological examination of tumor tissues from mice 28 days after T-01 inoculation. T-01 inoculations for 1 week or 4 weeks resulted in positive HSV-1 (Figure 5D and 5F), and HE staining showed tumor cell destruction in locations of virus amplification (Figure 5C and 5E). In addition, the pathology of the 4-week inoculation group revealed that HSV1-positive cells were more widely observed with the increasing number of virus administrations and stronger staining was observed in areas of cell destruction. In contrast, PBS inoculation for 4 weeks had no positive HSV-1 (Figure 5B) and HE staining showed no tumor cell destruction (Figure 5A).

**Figure 6:**
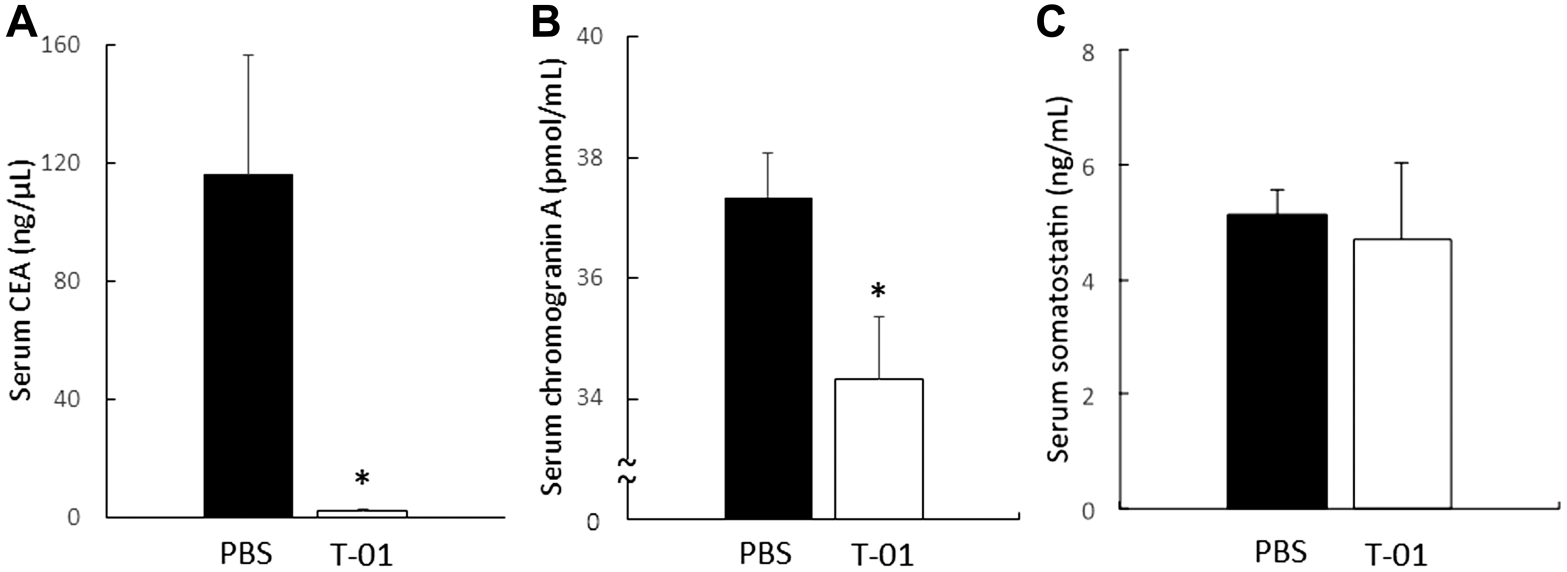
Serum CFA, chromogranin A and somatostatin in mice with subcutaneous tumors. QGP1-derived tumors in female athymic mice were inoculated at 1 and 3 days with T-01 (2 × 10^7^ pfu) or PBS. Mice were anesthetized 14 days after inoculation and blood was collected; serum CEA (**A**), chromogranin A (**B**) and somatostatin (**C**) were measured by ELISA. PBS infection (filled), T-01 (open). Data represent mean ± SE (*n* = 5). ^*^ *P* < 0.05 vs. PBS treatment.

**Figure 7:**
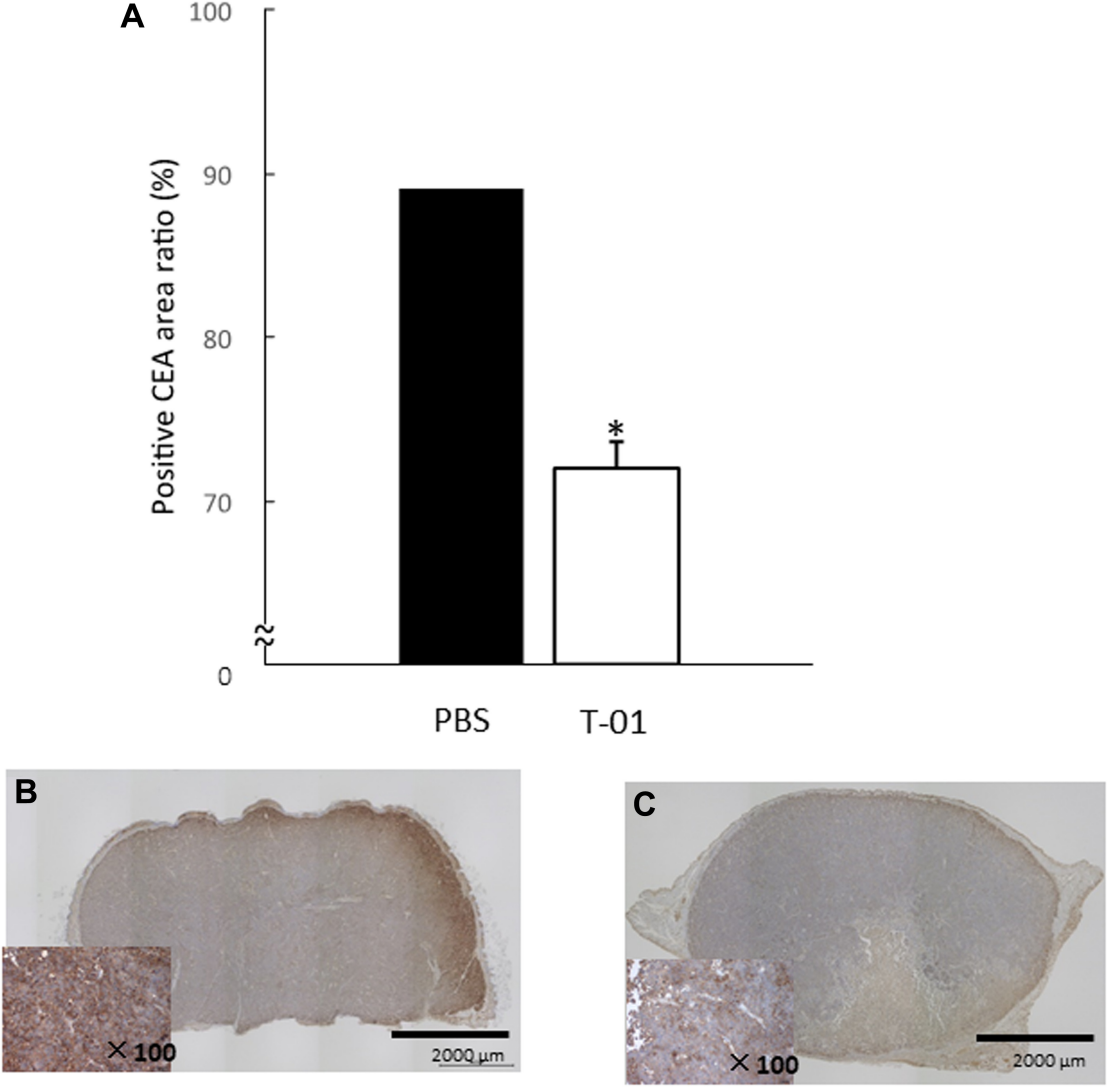
Quantitative analysis of intratumoral CEA in mice with subcutaneous tumors. QGP1-derived tumors in female athymic mice were inoculated twice (1 and 3 days) with T-01 (2 × 10^7^ pfu) or PBS. Mice were anesthetized 14 days after inoculation, and tumors were collected. Tissue sections were incubated with anti-CEA antibody. Positive CE areas were quantified (**A**) and representative images of PBS (**B**) and T-01 infection (**C**) are shown. Data represent mean ± SE (*n* = 5). ^*^ *P* < 0.05 vs. PBS treatment.

We also performed hematologic and histologic examination at a stage in which no difference in tumor diameter and body weight was observed with administration of T-01 (14 days, based on our unpublished observation). Blood and tumor samples were collected from mice in the T-01 and PBS groups (*n* = 5 per group). Serum CEA levels were suppressed in the T-01 group compared with the PBS group (Figure 6A). Serum chromogranin A was also suppressed in the T-01 group (Figure 6B). However, serum somatostatin did not show differences between the two groups (Figure 6C). Fewer CEA-positive cells in tumor tissues and blood were observed in the T-01 administration group in comparison with the PBS group (Figure 7). Together, these results suggest that the production of CEA is suppressed in both blood and tumor tissues in the T-01 administration group before differences in tumor growth occur.

## DISCUSSION

Gastroenteropancreatic neuroendocrine neoplasms with proliferation index (Ki67) > 20% and/or mitoses > 20 per 2 mm^2^ are aggressive tumors categorized in the Grade 3 group in the WHO classification of tumors of the digestive system, with an expected 5-year survival of 16% [20–22]. In a Nordic study of 305 NEC patients, the median overall survival (OS) was 11 months for patients treated with chemotherapy and 1 month for untreated patients [23]. Patients with pancreatic tumors showed a median OS of 15 months, while patients with rectal and colon tumors had a median OS of 10 and 8 months, respectively, indicating that OS differs with primary tumor location [24, 25]. Platinum-based combination chemotherapy with cisplatin/carboplatin and etoposide is the first-line treatment for gastroenteropancreatic neuroendocrine neoplasms [26, 27]. However, even with these treatments, the prognosis is poor, and effective secondary therapy has not been established when tumor progression is observed after initial treatment.

The primary goal of antitumor therapy is to specifically target tumor cells while protecting adjacent normal tissue from destruction. A previous report explored virus therapy for neuroendocrine tumors using adenovirus [28]. In the current study, we explored oncolytic virus as an alternative treatment approach to chemotherapy. Oncolytic virus therapy with a recombinant HSV-1 (T-01) is a treatment method in which cancer cells are destroyed by the direct cell-killing action of a virus and also by enhanced antitumor efficacy via T cell-mediated immune responses. The latter “T cell-mediated immune responses” rather enhanced under normal immune system, resulting in more strong oncolytic effects [29]. We previously reported that in a bilateral Hepa1-6 subcutaneous tumor model in C57BL/6 mice, the growth of tumors inoculated with T-01was inhibited, as was the case for contralateral tumors [19]. T-01 also reduced tumor growth on contralateral tumors. T-01 infection significantly enhanced antitumor efficacy via T cell-mediated immune responses.

T-01 used in this study has a cancer cell-selective replication ability due to deletion of γ34.5 gene and inactivation of ICP6 (a gene encoding ribonucleotide reductase). Since these are common properties in cancer cells, T-01 replicates selectively only in cancer cells. The oncolytic virus with genetic engineering modification replicates in cancer cells and then destroys host cancer cells. The increased progeny virus is scattered and infects cancer cells again and then repeats the replication → cell death → infection sequence to exert an antitumor effect. Notably, normal tissues are not damaged because the oncolytic virus that infects normal cells does not replicate.

In our study, we determined the *in vitro* cytopathic effects of the third generation oHSV T-01 in cell lines established from human/mouse neuroendocrine tumors (NET/NEC) and the possibility of oHSV T-01 treatment in a NEC (QGP1) mouse model. The *in vitro* and *in vivo* cytotoxicity and virus amplification results suggested that T-01 efficiently suppresses tumor growth in neuroendocrine tumors.

The STC-1 cell line is a neuroendocrine cancer cell line derived from a transgenic mouse, and the NCI-H727 cell line is a human endocrine neoplastic tumor cell line (NET G1/2). Because no study has yet reported a mouse subcutaneous transplantation model with STC-1 cells, we first attempted to create a STC-1 tumor model by performing subcutaneous transplantation of STC-1 cells (1.0 × 10^5^ to 1.0 × 10^8^) with or without Matrigel^®^ in C57BL6 mice with normal immunity. However, we were unable to successfully obtain a mouse subcutaneous transplantation model with STC-1 cells. In the current study, we used QGP-1 cells to investigate the use of T-01 virus treatment for neuroendocrine cancer originating from human pancreas (an intractable tumor) because a previous study [30] reported a subcutaneous transplantation model in nude mice using QGP-1 cells.

In the histological examination of the tumor sections in the T-01 administration group, the Ki-67 index was markedly high (49.8% to 86.4%) in the T-01 groups, indicating that cell proliferation ability was strong (data not shown). *In vivo* results with the QGP1 mouse model showed that T-01 exhibited dose-dependent and administration frequency-dependent inhibitory effects on tumor growth. The high Ki-67 index may have reflected the therapeutic effect of the virus.

We also observed that with increasing tumor growth, the mouse body weight decreased significantly in the PBS group. Many mice from the PBS group showed diarrhea, but no mice from the 4-week administration group showed diarrhea (data not shown). A previous study reported that QGP1 cells can produce somatostatin [30], and thus diarrhea and weight loss may be a symptom accompanying the tumor. These symptoms may be reduced when the tumor is smaller. Our results showed that tumor growth was suppressed by T-01 administration, and body weight loss was suppressed depending on T-01 dose and administration frequency. However, there was no significant difference in somatostatin levels in blood between the virus treatment and PBS groups.

CEA levels in the blood and tumor were measured, and lower levels were observed in the group treated with T-01 compared with the PBS group. After administration of T-01, the activity of tumor cells appeared to be suppressed earlier than the volume of T-01 treated tumors started to dissociate from control tumors. A previous study reported that QGP1 cell-derived tumors produce CEA, a characteristic of the cell line itself [30]. We also considered whether CEA was involved in the growth inhibitory effect of QGP1 tumors by T-01 administration. At a stage in which no difference in tumor diameter and body weight was observed with administration of T-01, we found that the levels of CEA in the blood and tumor decreased in the T-01 administration group, indicating a stimulatory effect of CEA production on tumor growth. We also examined chromogranin A and somatostatin immunostaining. Although a further decrease in the positive rate was observed in the T-01 administration group, the positive rates in the tumor cells were originally low, and there was no significant difference between the T-01 administration group and the PBS group in the statistical analysis (data not shown).

Human tumor cells infected with an α47-deficient HSV-1 express increased levels of MHC class I molecules and stimulate immune cells to a greater extent compared with uninfected cells [13]. Furthermore, in immunocompetent mice, oHSV inoculated into subcutaneous tumors was not detected in remote, non-inoculated tumors [31]. In our previous *in vivo* study, we examined T-01 administration in tumors from a hepatocellular carcinoma cell line [19]. Our study demonstrated that in immunocompetent mice bearing bilateral subcutaneous Hepa1-6 tumors, T-01 infection inhibited the growth of the contralateral non-inoculated tumor and inoculated tumor compared with the PBS group. Moreover, CD8^+^ splenocytes from mice primed with T-01 released higher levels of IFN-γ in response to Hepa1-6, indicating the increased number of lymphocytes that specifically recognized Hepa1-6 tumor cells. Furthermore, the numbers of CD8^+^ cells that infiltrated the Hepa1-6 tumors were significantly increased in the treated and untreated tumors of T-01-treated mice [19].

Oncolytic virus therapy has a therapeutic effect on distant metastasis of cancers through the induction of specific anti-cancer immunity in addition to the direct cancer cell destruction associated with viral replication. To elicit specific anti-cancer immunity, the virus must replicate in cancer cells. Cancer cells are destroyed when the immune system eliminates the virus and are processed into antigen-presenting cells together with the virus. We speculated that specific immunity against cancer cells occurs as a byproduct of the immune system process of eliminating the virus [13, 14].

In this study, we attempted to use STC-1 cells, which is a mouse-derived NEC cell line, in immunocompetent mice, but we found it difficult to establish a tumor model. So far, no reports are available on subcutaneous tumors or orthotopic models using immunocompetent mice for NET.

Regarding safety, oHSV harbors mutations in the viral genome that restrict viral replication to tumor cells and therefore oHSV kills host tumor cells without harming normal tissue [12]. Weight loss was not observed in the group of BALB/c nu/nu mice in which tumor growth was suppressed by administration of T-01, suggesting that T-01 is harmless to normal tissues. The T-01 virus used in this study is currently undergoing clinical trials in humans for brain tumors and prostate cancer [18].

In conclusion, our study demonstrated that T-01 effectively inhibited tumor cell proliferation in a poorly differentiated NEC mouse model. Oncolytic virus therapy using a third-generation HSV-1 may become a new therapeutic approach for NET that can be further combined with anticancer drugs or immune checkpoint inhibitors.

## MATERIALS AND METHODS

### Cell lines, viruses, and mice

The QGP1 human neuroendocrine cancer cell line was purchased from Research Bioresearch Collection, Japan (Osaka, Japan) [30]. A human NET cell line (NCI-H727) [32] and mouse NET cell line (STC-1) [33] were purchased from American Type Culture Collection. The African green monkey kidney (Vero) cell line was obtained from RIKEN BioResource Center (Tsukuba, Japan). Virus stocks were released from virus-infected Vero cells with heparin and then prepared by high speed centrifugation as previously described [18].

Five-week-old female athymic mice (BALB/c nu/nu) were purchased from Japan SLC, Inc. (Shizuoka, Japan) and used at 6 weeks of age. Mice were caged in groups of four or less. Animal care and experiments were performed in accordance with the standards outlined in the ARRIVE [34] and PREPARE [35] guidelines. Mouse studies were conducted according to guidelines approved by the Animal Care and Use Committee of Kansai Medical University (Approval no. 16-069, 17-016).

### *In vitro* cytotoxicity

*In vitro* cytotoxicity assay was performed as described previously [13, 29]. Cells were seeded in six-well plates, incubated overnight at 37°C and then inoculated for 1 h using virus or PBS. The medium was removed and the cells were incubated at 34.5°C in fresh medium supplemented with 1% FCS. The number of viable cells was counted daily using a Coulter Counter (Beckman Coulter, Fullerton, CA, USA) and expressed as a percentage of the mock infection control. The number of viable cells was consistent with that measured using trypan blue exclusion.

### *In vitro* virus yield

Cells (5 × 10^5^ cells/well) were seeded in 6-well plates and cultured for 24 h at 37°C. T-01 was then infected at a multiplicity of infection (MOI) = 0.01 and cells were incubated at 37°C for 48 h. After three freeze-thaw cycles, the cells were scraped and lysed. Titers of progeny virus stocks were determined using plaque assays with Vero cells. Each experiment was performed three times.

### Establishment of xenograft model and treatments

QGP1 cells (5 × 10^6^) were subcutaneously injected in the right flank of athymic mice. When the tumors reached a diameter of 5 mm, animals were randomly allocated into groups. T-01 virus (2 × 10^6^ or 2 × 10^7^ pfu) in 20 μL of PBS containing 10% glycerol was injected into tumors; the PBS group was injected only with PBS. In one experiment, PBS and T-01 groups that were injected with PBS or T-01 twice on day 0 and day 3 (two total inoculations) were compared (*n* = 8 per group). Tumor growth was monitored for 4 weeks after virus inoculation. Tumor size was measured using calipers, and tumor growth was determined by measuring the tumor volume (0.5 × (long axis) × (short axis)^2^) twice weekly. In the second experiment, virus concentration was kept constant at 2.0 × 10^7^ pfu. PBS and T-01 groups were injected with PBS or T-01 twice weekly for 1, 2 or 4 weeks (total of two, four or eight inoculations) (*n* = 8 per group).

Mice were sacrificed when the mice were in a moribund state (lethargy, supine or supine position, limited walking motion in response to rough respiration or stimulation) or if the maximum diameter of the tumor exceeded 20 mm. Mice were anesthetized by intraperitoneal injection of pentobarbital. Subcutaneous tumors were excised, fixed with formaldehyde and embedded in paraffin for histological analysis. This study followed the NIH Office of Animal Care and Use guidelines [31].

### Histochemical analysis

#### Hematoxylin-eosin (HE) staining and immunohistochemical analysis of HSV-1

Mice were sacrificed 28 days after infection with T-01 virus or PBS (two 2.0 × 10^7^ pfu or PBS injections on days 0 and 3) (*n* = 3 per group). Subcutaneous tumor tissues were embedded in 10% formalin and sections (5 μm thick) were placed on silanized slides (Dako Cytomation, Glostrup, Denmark) and stained with HE. Sequential tumor sections were cut and subjected to immunohistochemical analysis of HSV-1. Endogenous peroxidase activity was blocked to prevent nonspecific binding of secondary antibody. Samples were incubated with rabbit polyclonal anti-HSV-1 antibody (1:50000) (Dako Cytomation), rinsed and then incubated with goat anti-rabbit IgG antibody (Nichirei Bioscience Inc., Tokyo, Japan).

#### Hematological examination and evaluation of carcinoembryonic antigen (CEA) positive area

Hematological and histological examinations were performed 14 days after virus administration to investigate the influence of virus on blood and tumors. Tumors were inoculated with T-01 (2.0 × 10^7^ pfu, two inoculations, days 0 and 3) or PBS (*n* = 5 per group) and then observed for 14 days. No differences in tumor diameter and body weight were observed between T-01 and PBS groups. Mice were anesthetized and sacrificed. Blood was collected and tumors were harvested. Quantitative determination was performed using ELISA kits for serum levels of chromogranin A (Yanaihara Institute Inc., Shizuoka, Japan), somatostatin and CEA (RayBiotech, Inc. Norcross America, USA) according to the manufacturer’s instructions.

Immunostaining for CEA was performed on specimens prepared from paraffin blocks of subcutaneous tumor tissues. The CEA-positive area was calculated to determine the amount of CEA produced in the tumor and compared between the T-01 and PBS groups (*n* = 4 per group). Imaging was obtained using BZ-9100 (Keyence Corporation, Osaka, Japan), and the tumor area was analyzed using image analysis software (WinROOF Ver. 7.2.1; Mitani-shoji, Fukui, Japan). The area of the CEA-positive site was measured. The area within the range (total cross sectional area) was measured along the contour outer edge of the tumor part. The total area stained in brown by 3,3′-diaminobenzidine tetra-hydrochloride used as a chromogenic substrate was measured up to the first decimal place in units of 2 μm regardless of color intensity. The ratio of positive CEA area (%) to the tumor area was calculated.

### Statistical analysis

Data are presented as mean ± standard error. *In vitro* data and *in vivo* tumor volume data were evaluated using Student’s *t*-test. The statistical significance for the comparison of multiple sample sets was determined with the Tukey–Kramer test. Statistical significance was defined as *P* < 0.05. Statistical analysis was performed with the R version 3.4.3 (R Foundation for Statistical Computing, Vienna, Austria).
